# Varoglutamstat: Inhibiting Glutaminyl Cyclase as a Novel Target of Therapy in Early Alzheimer’s Disease

**DOI:** 10.3233/JAD-231126

**Published:** 2024-10-18

**Authors:** Howard H. Feldman, Karen Messer, Yuqi Qiu, Marwan Sabbagh, Douglas Galasko, R. Scott Turner, Oscar Lopez, Amanda Smith, January Durant, Jody-Lynn Lupo, Carolyn Revta, Archana Balasubramanian, Kerstin Kuehn-Wache, Tanja Wassmann, Sylvia Schell-Mader, Diane M. Jacobs, David P. Salmon, Gabriel Léger, Mari L. DeMarco, Frank Weber, for the ADCS VIVA-MIND Study Group

**Affiliations:** a Department of Neurosciences, University of California San Diego, La Jolla, CA, USA; b Alzheimer’s Disease Cooperative Study, University of California San Diego, La Jolla, CA, USA; c Shiley-Marcos Alzheimer’s Disease Research Center, La Jolla, CA, USA; d Department of Statistics, East China Normal University, Shanghai, China; e Department of Neurology, Barrow Neurological Institute, Phoenix, AZ, USA; f Department of Neurology, Georgetown University, Washington, DC, USA; g Department of Neurology, University of Pittsburgh, Pittsburgh, PA, USA; h Department of Psychiatry, University of Pittsburgh, Pittsburgh, PA, USA; i USF Health Byrd Alzheimer’s Institute, Tampa, FL, USA; j Vivoryon Therapeutics NV, Halle, Germany; k Department of Pathology and Laboratory Medicine, Providence Health Care, Vancouver, BC, Canada; l Department of Pathology and Laboratory Medicine, University of British Columbia, Vancouver, BC, Canada

**Keywords:** Alzheimer’s disease, amyloid β-peptides, CCL2, cerebrospinal fluid, glutaminyl cyclase, mild cognitive impairment, N3pE-Aβ, pGlu-Aβ, QPCT, QPCTL

## Abstract

**Background:**

Varoglutamstat is a first-in-class, small molecule being investigated as a treatment for early Alzheimer’s disease (AD). It is an inhibitor of glutaminyl cyclase (QC), the enzyme that post-translationally modifies amyloid-β (Aβ) peptides into a toxic form of pyroglutamate Aβ (pGlu-Aβ) and iso-QC which post-translationally modifies cytokine monocyte chemoattractant protein-1 (CCL2) into neuroinflammatory pGlu-CCL2. Early phase clinical trials identified dose margins for safety and tolerability of varoglutamstat and biomarker data supporting its potential for clinical efficacy in early AD.

**Objective:**

Present the scientific rationale of varoglutamstat in the treatment of early AD and the methodology of the VIVA-MIND (NCT03919162) trial, which uses a seamless phase 2A-2B design. Our review also includes other pharmacologic approaches to pGlu-Aβ.

**Methods:**

Phase 2A of the VIVA-MIND trial will determine the highest dose of varoglutamstat that is safe and well tolerated with sufficient plasma exposure and a calculated target occupancy. Continuous safety evaluation using a pre-defined safety stopping boundary will help determine the highest tolerated dose that will carry forward into phase 2B. An interim futility analysis of cognitive function and electroencephalogram changes will be conducted to inform the decision of whether to proceed with phase 2B. Phase 2B will assess the efficacy and longer-term safety of the optimal selected phase 2A dose through 72 weeks of treatment.

**Conclusions:**

Varoglutamstat provides a unique dual mechanism of action addressing multiple pathogenic contributors to the disease cascade. VIVA-MIND provides a novel and efficient trial design to establish its optimal dosing, safety, tolerability, and efficacy in early AD.

## INTRODUCTION

Varoglutamstat [(+)-(S)-1-(1H-benzo[d]imidazol-5-yl)-5-(4-propoxyphenyl) imidazolidin-2-one and its hydrochloride, also known as PQ 912] is a first-in-class, highly specific and potent small molecule with a unique dual mechanism of action targeting amyloid-β (Aβ) and neuroinflammation. It is being investigated as a treatment for early Alzheimer’s disease (AD). This paper aims to a) review the scientific rationale and mechanism of action of varoglutamstat, b) set the context for the varoglutamstat development program, and c) present the design and methodology of the “VIVA-MIND” clinical trial.

### Therapeutic targets

**Fig. 1:**
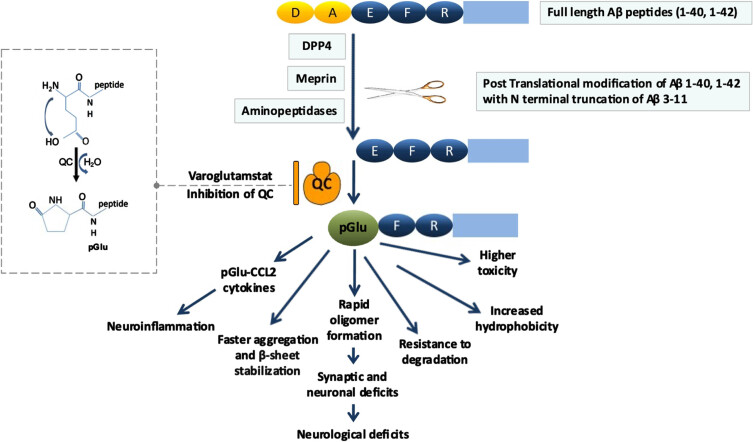
pGlu-Aβ as a therapeutic target^a^. Aβ is cleaved by dipeptidyl peptidase 4 (DPP4), meprin β, or aminopeptidases, between residues 2 (alanine [A]) and 3 (glutamate [E]). This exposes glutamate at the N-terminus, which is subsequently modified to N-terminal pyroglutamate (pGlu) by dehydration catalyzed by glutaminyl cyclase (QC) activity. The resultant peptide (pGlu-Aβ) has altered biochemical properties with severe pathological consequences. The enhanced toxicity is likely due to the higher aggregation propensity and the longer bioavailability of the pGlu-Aβ oligomers. Additionally, an isoenzyme of QC converts the N-terminus of chemokine ligand 2 (CCL2) into pGlu-CCL2 which is associated with neuroinflammation. Varoglutamstat inhibits both QC and iso-QC. ^a^Figure and caption are adapted from Jawhar, Wirths, and Bayer (2011) (CC BY 4.0 DEED), https://creativecommons.org/licenses/by/4.0/#

Varoglutamstat’s therapeutic targets are post-translationally modified pyroglutamate amyloid-β (pGlu-Aβ, also known as pGlu-Aβ 3-42, N3pE-Aβ) and post-translationally modified cytokine monocyte chemoattractant protein-1 (pGlu-CCL2). As illustrated in Fig. 1, varoglutamstat inhibits the enzyme glutaminyl cyclase (QC, also known as QPCT) and its isoenzyme iso-QC (also known as QPCTL) resulting in reduced levels of pGlu-Aβ, a post-translationally modified form of Aβ (Glutamate 3/11 cyclization), as well as pGlu-CCL2, a post-translationally modified form of CCL2 (Glutamine cyclization). These post-translationally modified proteins are significantly overexpressed in AD where pGlu-Aβ has been shown to be synaptotoxic, proinflammatory, promoting of self-aggregation into oligomers, and resistant to degradation [1], while pGlu-CCL2 has been linked to the presence and severity of neuroinflammation [2]. Evidence is accruing that these pGlu targets and pathways are independent of the therapeutic targets that have been tested with BACE 1 inhibition as well as Aβ_1-42_ directed monoclonal antibodies [3, 4].

### Scientific rationale for QC and iso-QC inhibition

In AD, there is increased QC mRNA expression in affected entorhinal cortex and cortical areas that co-localizes and correlates with pGlu-Aβ deposits and decline in cognitive function [5]. There is no corresponding correlation between QC mRNA and Aβ_1-40_ or Aβ_1-42_ peptides [5]. In human embryonic kidney (HEK) 293 cell lines with amyloid-β protein precursor (AβPP) overproduction, QC is needed for pGlu-Aβ production, an effect that is suppressible with QC inhibition [6]. Similarly in a transgenic (Tg) mouse, crossing 5XFAD with an overexpressing human QC model (5XFAD/hQC) leads to increased pGlu-Aβ and phenotypic impairment including deficits in working memory and motor function at 6 months of age [7]. These effects are not seen in 5XFAD mice alone. Conversely, a Tg 5XFAD×QC knockout rescues the wild type behavioral phenotype implicating QC in pGlu-Aβ formation and its regulation [7]. Furthermore, treating Tg2576 mice (APP K670N and M671L mutations) with long term QC inhibition from 6 months to 16 months strongly reduces the amount of pGlu-Aβ in a dose dependent manner and decreases Aβ_x-42_ and Aβ_x-40_, with diminished formation of cortical plaques and plaque associated immunoreactive astrocytes and microglia [8]. Behaviorally, QC inhibition in Tg2576 mice is associated with improvement in conditioned fear learning that requires context memory [8].

In AD, the severity of neuroinflammation with activation of microglia and astrocytes correlates with cognitive decline and predicts brain atrophy [9]. Among the cytokines and chemokines, CCL2 is a strong predictor of AD with a dominant role in the chronic inflammatory process [2, 10]. In both aged APP Tg2576 mice and in human AD, iso-QC and CCL2 mRNA co-localize and are upregulated in the presence of Aβ peptides [11]. In primary mouse astrocyte cultures, both are found with only very weak immunocytochemical signal until induced with Aβ or pGlu-Aβ when they increase robustly [11]. CCL2 overexpression within a Tg mouse model (Tg2576swe×CCL2) accelerates the formation of Aβ oligomers and diffuse plaques (by 5X compared to APP alone), while increasing activated monocyte derived macrophage and microglia accumulation that is associated with accelerated memory impairment [12–14].

### Pharmacokinetic and pharmacodynamic properties of varoglutamstat

The pharmaceutical properties of varoglutamstat, including pharmacokinetic and pharmacodynamic effects, have been evaluated in numerous preclinical assays and models. In HEK 293 cellular models, it has higher potency in inhibiting the cyclization of glutamate compared to glutamine providing target selectivity to QC inhibition [15]. Across species including mice, rats, and humans, varoglutamstat has potent QC Ki values between 20 and 65 nM [15]. In the Tg animal model hAPPSLxhQC, an oral dose of 0.8 g/kg (200 mg/kg/day) produced a significant reduction in pGlu-Aβ and a significant improvement in spatial learning and memory in the Morris Water Maze. Short term 3-week treatment with varoglutamstat in hAPPslxhQC mice was associated with improvement in spatial learning without detectable reduction in pGlu-Aβ. Longer term treatment of 4 months reduced both soluble and insoluble pGlu-Aβ [15]. In a satellite experiment, levels of (free) varoglutamstat in cerebrospinal fluid (CSF) and brain were determined sequentially over a period of 24 h after 1-week of treatment via chow containing 0.8 g/kg. Mean drug concentration in CSF was about 15 ng/ml which indicates a QC inhibition of 60% in CSF (TO (%) = 100*C/(Ki+C); TO = target occupancy in %, Ki = Inhibitory constant of varoglutamstat = 25 nM for human QC, and C = measured concentration of varoglutamstat in CSF) [15]. In line with these results are findings from QC KO experiments showing that a robust therapeutic effect required QC inhibition of more than 50% to achieve significant reduction of pGlu-Aß and concomitant behavioral improvement [7]. This ability to define a translational threshold of enzyme target occupancy (TO) is important in selecting the dose range for human trials.

### Prediction of clinically effective doses

**Fig. 2:**
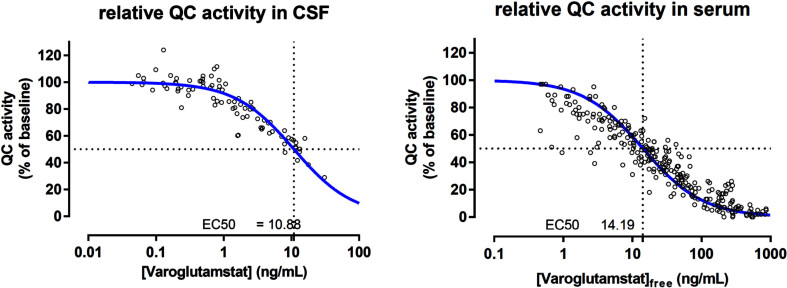
The pharmacokinetic/pharmacodynamic relationship between varoglutamstat concentration and QC inhibition in CSF and serum^a^. QC, glutaminyl cyclase; CSF, cerebrospinal fluid; ng, nanogram; mL, milliliter. ^a^Data on file with Vivoryon Therapeutics.

Animal data indicate that the therapeutic target inhibition level is 50% or higher, and this result is used to estimate potential effective doses in patients. A threshold value of a mean CSF drug concentration of about 10–15 ng/ml achieves both sufficient inhibition of pGlu-Aβ formation and behavioral response in AD mice. The dose-dependent area under the curve (AUC) of varoglutamstat in CSF has been determined in a phase 1 multiple ascending dose (MAD) study with TO calculated based on the same formula as described above [16]. In CSF from elderly subjects, 200 mg BID of varoglutamstat led to an AUC of 16 ng/ml relating to a mean TO of 62%. Higher doses of 300, 500, and 800 mg BID led to varoglutamstat concentrations in CSF which were equivalent to mean TO of 71, 84, and 91%, respectively, resulting in a sigmoid function for QC inhibition/target occupancy versus dose. In good agreement with the calculation of TO was the result of the PK/PD relationship established in phase 1 between varoglutamstat concentration and degree of inhibition of QC-activity in CSF seen in Fig. 2 [17]. This relationship was characterized by an EC50 value of 10.9 ng/ml (30 nM), which is about the same as the Ki value for the isolated QC enzyme (25 nM) that was estimated at the isolated enzyme and used to calculate TO.

### Human development

Varoglutamstat has successfully completed phase 1 and initial phase 2A clinical trials. In phase 1, a single ascending dose (SAD) study included 83 healthy volunteers aged between 22–55 years who received doses from 10 mg–3600 mg. In MAD studies of 11 days duration, 47 participants aged 22–55 years received doses of 20–500 mg in fasted and fed states, and 32 participants aged 65–77 years received 200–800 mg BID doses [17]. In the older MAD subgroup, there was a 1.5–21-fold increase in AUC over 0–12 h and Cmax compared to the younger group. Cmax in CSF was reached in 2–3 h while plasma t max was reached in 0.5–1.0 h. CSF/plasma AUC was independent of dose. In addition to establishing the safety of this wide dose range, CSF sampling showed concentrations with mean TO of 90% in CSF for 800 mg BID, the dose selected for the first phase 2A study [17].

Most treatment emergent adverse events (TEAEs) in phase 1 were mild or moderate in severity and resolved without treatment. Gastrointestinal TEAEs were the most frequent with similar incidence across dose groups excepting a higher incidence for the two highest dose levels among older participants [17]. The most frequent single preferred term TEAE was headache. Among the few severe adverse events (TESAEs), there was an individual with urticarial papular rash that resolved [17]. There were no lab safety findings considered to be clinically significant and no changes in vital signs or EKG [17].

The initial phase 2A study, SAPHIR (NCT02389413), investigated the safety, tolerability, and efficacy of varoglutamstat in 120 treatment-naïve participants with mild cognitive impairment (MCI) or mild dementia due to AD [16]. Participants were treated for 12 weeks with varoglutamstat (first week: 400 mg BID, thereafter 800 mg BID) or placebo. Participants treated with varoglutamstat reported more TEAEs (135) and TESAEs (13) than placebo (103 TEAEs, 3 TESAEs), particularly gastrointestinal and skin and subcutaneous disorders. In the varoglutamstat group, 26 participants (43.3%) did not adhere to the treatment and 20 of these discontinued the study due to AEs. The majority of reported safety and tolerability events started between treatment weeks 3 and 8, with very few new events between weeks 8 and 12. While the 800 mg BID dose achieved the predicted average TO of > 90%, there were significant differences on primary composite endpoints of safety and tolerability, indicating the dose range would need to be lowered in further phase 2 trials. Importantly, no relevant AEs were reported during the first week of the SAPHIR trial when participants were receiving 400 mg BID [16]. Results on exploratory efficacy measures indicated significantly reduced theta power on EEG spectral analyses. Theta power is known to increase with AD progression and varoglutamstat had the intended effect of lessening this increase [16, 18]. There were preliminarily favorable findings showing CSF YKL-40 decreased approximately 5% and Neurogranin (NRGN) decreased approximately 4% [16].

There are currently two ongoing phase 2 clinical trials evaluating varoglutamstat in people with AD: 1) VIVIAD and 2) VIVA-MIND. The VIVIAD study (NCT04498650) [19] is a phase 2B multicenter, randomized, double-blind, placebo-controlled, parallel group dose finding, safety, tolerability, and efficacy study of varoglutamstat in subjects with MCI and mild dementia due to AD. As of July 2023, the trial is fully enrolled with 259 participants and an estimated study completion date in the first quarter of 2024.

The VIVA-MIND study is a phase 2A-B randomized, double-blind, placebo-controlled trial to evaluate the efficacy and safety of varoglutamstat in patients with early AD. VIVA-MIND includes a “seamless” design where phases 2A and 2B run without interruption. The objective of phase 2A is to determine the highest dose that is both safe and well tolerated. During this phase, continuous safety evaluation using a well-defined safety stopping boundary determines which dose will be carried forward in phase 2B. There is a planned Stage Gate between phase 2A and 2B. Following a successful outcome of the Stage Gate, phase 2B will assess the longer-term efficacy and safety of varoglutamstat using the phase 2A selected highest dose. The therapeutic hypothesis is that through the inhibition of the two enzymes (QC and iso-QC), with reduction of pGlu-Aβ and CCL2, there will be lessening of Aβ related toxicity and disease modification. In the following sections we focus on the VIVA-MIND clinical trial, elaborating details of this seamless phase 2A-2B design to test the efficacy and safety of varoglutamstat in the treatment of early AD.

## MATERIALS AND METHODS

### Study setting and design of VIVA-MIND

**Fig. 3:**
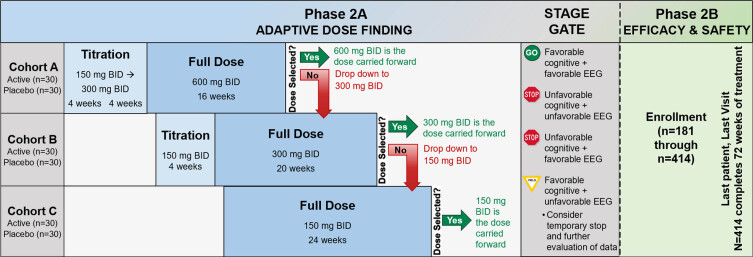
Study schematic. BID, twice per day; mg, milligram; EEG, electroencephalogram.

The study schematic is presented in Fig. 3. In phase 2A, 180 participants will be randomized using a 1:1 allocation to active treatment or placebo. Following phase 2A, an interim futility analysis will be undertaken with a Stage Gate decision for whether to continue to phase 2B. In phase 2B, there will be enrollment of 234 additional participants, using the same inclusion criteria as phase 2A, to a total sample size of 414. These newly-enrolled phase 2B participants will be randomized 1:1 to active treatment or placebo. The recruitment and enrollment of participants is occurring through sites of the Alzheimer’s Disease Cooperative Study network, with 22 clinical sites in phase 2A and a planned 55 clinical sites in phase 2B. The study was registered at ClinicalTrials.gov (NCT03919162) on April 18, 2019 and the protocol has received central Institutional Review Board approval from Advarra. The Investigational New Drug application to the U.S. Food and Drug Administration received approval on July 31, 2020 (IND143871) and is the responsibility of Vivoryon Therapeutics AG.

### Study population

VIVA-MIND includes male and postmenopausal or surgically sterile females, aged 50 to 89 years, who meet criteria for MCI due to AD or mild probable AD according to National Institute on Aging and Alzheimer’s Association (NIA-AA) Diagnostic Guidelines [20, 21] and have a CSF biomarker profile consistent with AD pathology: Aβ_1-42_ < 1030 pg/mL AND tau phosphorylated at threonine 181 (p-tau-181) > 27 pg/mL, OR p-tau-181/Aβ_1-42_ > 0.023 (Roche Elecsys® assays) [22, 23]. Other key inclusion criteria are the following scores at screening: Mini-Mental State Examination (MMSE) [24] score 20–30 (inclusive), Montreal Cognitive Assessment (MoCA) [25] score < 26, and Clinical Dementia Rating (CDR) [26] global score of 0.5 or 1.

Participants treated with acetylcholinesterase inhibitors and/or memantine are included if they have been on a stable dose for at least 4 months prior to screening and are expected to remain on a stable dosage regimen for the duration of the trial. Participants being treated with lecanemab (Leqembi^TM)^, aducanumab (Aduhelm^TM^), or any anti-amyloid monoclonal antibody (AAMA) are not eligible for inclusion. Varoglutamstat is a moderate inhibitor of the CYP2C19 enzyme; as such, the concomitant administration of strong inhibitors or inducers of the CYP2C19 enzyme or substrates with a narrow therapeutic margin are not permitted (e.g., fluconazole, fluvoxamine, lansoprazole, ticlopidine, rifampin, s-mephenytoin, phenobarbital, indomethacin) and concomitant administration with all other moderate or weak inhibitors or products predominantly metabolized through CYP2C19 should be done with caution.

### Study procedures, flow, and endpoints

Screening procedures to determine eligibility are completed after informed consent and include medical history, physical and neurological exam, MMSE, MoCA, CDR, blood collection, urinalysis, cranial MRI, resting 12-lead ECG, lumbar puncture, Columbia Suicide Severity Rating Scale (C-SSRS) [27], and the Modified Hachinski Ischemic Scale. Following screening determination of eligibility, participants are randomized and undergo baseline procedures for primary, secondary, and exploratory outcome measures. Additional study visits are completed at weeks 4, 8, 12, 16, 24, 36, 48, 60, and 72 with a final safety visit at week 76 (see Schedule of Events in Supplementary Material). A schematic of the flow of participants through the study is presented in Fig. 3.

*A) Phase 2A: Adaptive dose finding*

Phase 2A participants are enrolled sequentially into one of three dose cohorts with the first 60 subjects enrolled in cohort A (600 mg BID), the next 60 in cohort B (300 mg BID), and the last 60 in cohort C (150 mg BID). Subjects are randomized 1:1 to active drug or placebo within each dose cohort; there is a titration period for the two higher dose levels. Within each dose cohort there is a continuously monitored sequential Pocock safety boundary [28], which counts the number of adverse events of special interest (AESIs) among the subjects on active treatment, during the first 8 weeks each subject is on full dose. The Pocock sequential boundary defines the unacceptable number of participants (in the active arm) with an AESI during the first 8 weeks at full dose. From the SAPHIR study, AESIs have been defined as being within the MedDRA system organ class of skin and subcutaneous tissue disorders and hepatobiliary disorders. A dose will be discontinued if the dose cohort hits the stopping boundary. Subjects in that cohort will be down-titrated to the next available dose. The first dose to survive the full 8-week observation period will be the dose selected by phase 2A. As soon as a dose has been selected, all phase 2A participants (*n* = 180) will be titrated (or randomized) to the selected dose and treated for up to 72 weeks. The number of pills assigned to each participant irrespective of dose cohort or group is the same throughout, while the dose may change.

To summarize, the dose adaptive phase includes four possible scenarios: 1) If the selected dose is 600 mg BID (dose cohort A), then dose cohorts B and C will be titrated up to this dose; 2) If the selected dose is 300 mg BID (dose cohort B), all cohort A participants who are still receiving study medication (including those that did not experience an AESI and tolerated their assigned dose) will be reduced to 300 mg BID and dose cohort C will be titrated to 300 mg BID (participants in dose cohort C must have received at least 4 weeks of 150 mg BID before increasing their dose to 300 mg BID); 3) If the selected dose is 150 mg BID (cohort C), all participants in both cohorts A and B who are still receiving study medication (including those that did not experience an AESI and tolerated their assigned dose) will be reduced to 150 mg BID; 4) If cohort C meets the stopping rule, then the trial is halted.

*B) Interim futility analysis and Stage Gate to phase 2B*

The interim futility analysis will be conducted once participant number 180 reaches 24 weeks on full dose. The analysis will use all data available from phase 2A participants up to that point. The primary endpoints to determine futility are 1) within-participant change from baseline to week 24 on a cognitive composite outcome comprised of nine measures from the ADNI-1 [29] neuropsychological test battery (ADNI Battery Composite, ABC) compared between active and placebo arms, and 2) within-participant change from baseline to week 24 in a quantitative electroencephalogram (qEEG) measure (global relative theta wave power) compared between active and placebo arms. The use of EEG spectral analysis in VIVA-MIND builds on evidence that it may provide a sensitive indicator of pharmacodynamic effects which can add biologic evidence to drug reaching the brain and having a positive effect by preserving electrophysiologic function and reducing EEG patterns of progressive abnormalities [16, 30].

The ADNI neuropsychological test battery was selected because it provides a brief yet comprehensive cognitive assessment across multiple cognitive domains. Its publicly available data in an early-AD cohort has enabled the modeling of expected effect sizes for VIVA-MIND. This ABC composite is calculated by summing the standardized scores on the following 9 measures from the ADNI-1 neuropsychological test battery: Rey Auditory Verbal Learning Test Immediate Recall (Trials 1-5); Rey Auditory Verbal Learning Test Delayed Recall; Number Span Forward (Number of Correct Spans); Number Span Backward (Number of Correct Spans); Category Fluency (average of Animal and Vegetable Fluency); Trail Making A, Time to Completion (negative of score); Trail Making B, Time to Completion (negative of score); WAIS-R Digit Symbol Substitution, Total Correct; Boston Naming Test (30 item), Total Correct. Each measure is scored so that higher is better (i.e., the negative value will be taken for tests where higher is worse, as indicated). For each subject and each test, the test score is standardized by subtracting the overall baseline mean and dividing by the baseline standard deviation. The baseline mean and standard deviation statistics for each measure is computed using the entire enrolled study population. Then the ABC score for each subject is the sum of the standardized test values.

**Fig. 4:**
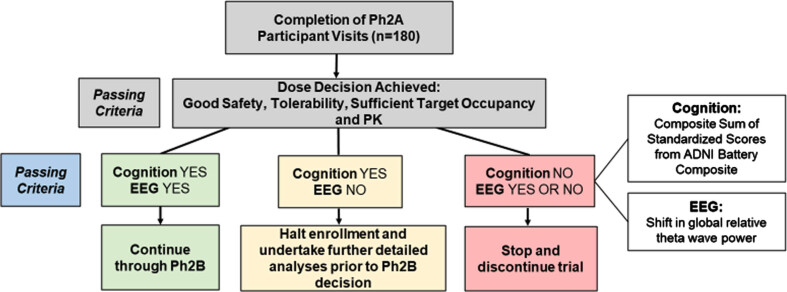
Stage Gate framework for proceeding to phase 2B. Ph2A, phase 2A; EEG, electroencephalogram; PK, pharmacokinetic; ADNI Battery Composite, Alzheimer’s Disease Neuroimaging Initiative sum of 9 standardized cognitive test scores.

As shown in Fig. 4, if the analysis shows evidence of negative cognitive effects on the ABC measure (Cognition NO), the trial will stop. If there is no evidence of a negative cognitive effect (Cognition YES) and evidence of benefit on EEG theta power (EEG YES), the trial will continue. If there is no evidence of a negative cognitive effect (Cognition YES) and no evidence of benefit on EEG theta power (EEG NO), the stopping rule will be indeterminate, the trial will pause and further analysis may be undertaken to inform a final decision on continuation of the trial by the Study Steering Committee and study sponsor.

Secondary safety and tolerability endpoints will also be examined. These are: rates of all AEs, drug discontinuation rates, mortality rates, suicidality scores on the C-SSRS, significant changes on brain MRI scans (e.g., ARIA-E, ARIA-H, infarcts), frequency and severity of abnormality on physical exams, vital signs, health status, ECG, and safety labs.

In addition, PK sampling will be undertaken during both phases of the trial. During phase 2A samples will be drawn for determination of mean plasma varoglutamstat levels for each cohort following at least 8 weeks of treatment at the dose levels being tested. These PK samples will also be measured pre-and-post-dose starting at week 4 and every 4–8 weeks throughout phase 2A.

*C) Phase 2B*

Phase 2B will follow according to the outcome of the phase 2A Stage Gate decision. Newly-enrolled participants in phase 2B will receive the selected dose of varoglutamstat (or placebo) with its titration procedure carried forward from phase 2A.

The primary efficacy endpoint in phase 2B is the within-participant change in CDR Sum of Boxes (CDR-SB) from baseline to week 72. CDR-SB has shown good reproducibility across multiple studies as well as more robust placebo arm decline than other measures in the MCI and mild AD dementia population [31]. The key secondary efficacy endpoint in phase 2B is the within-participant change from baseline to week 72 on the Cognitive Function Composite 2 (CFC2) measure [31]. CFC2 was the best performing endpoint in a recent study of longitudinal cognitive change in MCI and early AD [31]. Other secondary efficacy endpoints include the within-participant change from baseline to week 72 on the ABC cognitive measure, the qEEG measure (global relative theta wave power), the Functional Activities Questionnaire (FAQ) [32], the ADAS-Cog13, and the Neuropsychiatric Inventory (NPI) [33].

PK of varoglutamstat in phase 2B will be measured in plasma at weeks 48 and 72. TO will be measured in both plasma and CSF at study endpoint. In addition, the ratio of plasma to CSF varoglutamstat will be determined.

Safety and tolerability endpoints in phase 2B are rates of all AEs, drug discontinuation rates, mortality rates, suicidality scores on the C-SSRS, changes on brain MRI scans (ARIA-E, ARIA-H, infarcts), frequency and severity of abnormality on physical exams, vital signs, health status, ECG, and safety labs.

Exploratory endpoints include the within-participant change in brain volume measured by cranial MRI, MMSE scores, MoCA scores, CSF biomarker measures (Aβ_1-42_, t-tau, p-tau-181, sTREM2, YKL-40, neurogranin, SNAP-25, NfL, and VILIP-1), qEEG connectivity network measures, the AD Composite Score (ADCOMS) [34], and the ADAS-Cog-Exec score [35]. Relative change from screening to week 72 in QC activity in CSF, and changes in the primary outcome measure (CDR-SB), key secondary outcome measure (CFC2), and the TO measure will be examined in subgroups defined separately by APOE genotype (*ɛ*4 carrier versus non *ɛ*4 carrier) or severity of cognitive impairment (MCI versus mild probable AD). Additional exploratory analyses in phase 2B include a comparison of plasma and CSF biomarkers of amyloid pathology. For plasma, testing is performed using PrecivityAD®, which uses a combination of the ratio of Aβ_1-42_ to Aβ_1-40_ along with APOE *ɛ*4 carrier status. For CSF, testing is performed using the Elecsys® Aβ_1-42_ and p-tau-181 assays.

### Statistical considerations

*Adaptive dose phase:* The stopping boundary was computed using an exact binomial distribution [28]. If all 3 doses have an acceptable AESI rate of 2.5% or less, as expected, the top dose will be selected with > 95% probability. In this case, the 600 mg dose would be selected within 16 weeks of randomization of patient number 60. Alternatively, if any dose has an unacceptable AESI rate of 20%, that cohort will stop early with probability 90%; the expected number of subjects treated on that dose is 15.

*State gate interim analysis:* The futility analysis is conducted using a one-sided test of hypothesis for each measure, using a mixed model for repeated measures (MMRM) with linear time trend and all available data from the phase 2A modified-intent-to-treat population. For the ABC cognitive measure, the null hypothesis is of benefit, and the alternative hypothesis is of harm from active treatment, tested at 40% significance level. This has been selected to identify early negative cognitive effects within the interim analysis. Under this test, if the treatment arm is 20% worse than control at 24 weeks, the trial will have 60% probability to stop at the interim analysis; if the treatment arm is 40% better than control, then there is 90% probability of a favorable outcome of the test. This guards against prolonged exposure in the face of negative cognitive effects.

For the EEG measure, the null hypothesis is of no benefit, and the alternative is of benefit. This test has 90% probability of a favorable outcome assuming the underly effect sizes are comparable to estimates from the SAPHIR trial. Hence, under favorable assumptions on each outcome, and assuming independence, the interim futility analysis has greater than 80% chance of continuing the trial. If, on the other hand, there is no underlying cognitive benefit to subjects at 24 weeks, but the theta power effect is similar to the SAPHIR trial, the interim analysis has more than 54% power to continue. If there is no difference between arms on EEG theta power, so that prior results from the SAPHIR trial do not replicate, then there is only 5% chance that the EEG hypothesis test will return a YES. If there is cognitive benefit as assumed above, the cognitive hypothesis test has 90% chance to return YES. Hence under this scenario, there is about 80% chance of an indeterminate result from the stopping rule.

*Sample size for phase 2B:* With approximately 207 participants per arm, there will be at least 80% power to detect a change of 0.7 points in CDR-SB, which is equal in magnitude to about 37% of the decline observed at 18 months in a similar observational study population in ADNI [31]. This assumes a mean change in CDR-SB at 72 weeks of about 1.9 points (SD 2.28 points) in the placebo arm, and a 40/60 mixture of MCI (mean change 1.2 points) to mild AD dementia participants (2.3 points) [31]. The calculation assumes no more than a 25% dropout rate over the study period.

*Analysis populations* are defined as: 1) a Safety population that includes all randomized participants who took at least one dose of the study drug, 2) an Intent-to-Treat (ITT) population that includes all randomized participants, 3) a Modified Intent-to-Treat (mITT) population for each efficacy measure that includes all randomized participants who took at least one dose of the study drug and have both a baseline assessment and at least one follow-up assessment of the measure, and 4) a Pharmacokinetic (PK) population that includes all randomized participants who have at least one PK sample timepoint.

## DISCUSSION

pGlu-Aβ has emerged as a compelling therapeutic target in AD. Once this post-translationally modified form of Aβ is formed, it becomes synaptotoxic and proinflammatory. It promotes its self-aggregation into oligomers and is resistant to degradation.

Donanemab, a humanized immunoglobulin G1 monoclonal antibody directed at N-terminal pGlu-Aβ epitope in amyloid plaques, both reduces pGlu-Aβ and clears amyloid plaques. This treatment has now been clinically validated with positive clinical trial and biomarker endpoints in its phase 2 and phase 3 trials [36, 37]. While donanemab has been generally well tolerated it requires parenteral infusion, with the potential for infusion reactions as well as amyloid related imaging abnormalities (ARIA) including edema and hemorrhage. In the recently completed phase 3 trial, 8.7% of participants receiving donanemab reported infusion reactions, 36.8% had either ARIA of edema/effusion or microhemorrhages and hemosiderin deposits, 6.1% had symptomatic edema or effusions [37]. Donanemab’s regulatory evaluation is currentlyongoing.

Varoglutamstat provides a novel first-in-class small molecule approach to reducing the production of post-translationally modified pGlu-Aβ and pGlu-CCL2. Its dual MOA inhibits the formation of pGlu-Aβ by inhibiting QC and modulates the neuroinflammatory elements of pGlu-CCL2 by inhibiting iso-QC [8, 12]. While varoglutamstat is similar to donanemab in lowering pGlu-Aβ, it differs in that it does not reduce aggregated Aβ through microglial mediated removal of cerebral amyloid plaques, and shows no signs of inducing ARIA. Whereas a BACE 1 gene knockout does not affect the levels of soluble and insoluble pGlu-Aβ in APP Tg mice, preclinical models that overexpress the QC enzyme, the protease peptidase cathepsin B and the endoprotease meprin, are associated with increased formation of pGlu-Aβ [3, 4, 38, 39]. Furthermore, by not directly targeting the production and clearance of endogenously produced Aβ_1-42_, there is no perturbation of its potential contribution to antimicrobial activity, tumor suppression, support of the blood-brain barrier, or regulation of synaptic function [40]. In contrast to AAMAs, which must be administered intravenously, varoglutamstat is a small molecule that can be delivered orally, with sufficient enzyme TO to establish target engagement within a dose range that is projected to have sufficient safety and tolerability. Thus, if the trials are positive, varoglutamstat should have fewer barriers for patient access and affordability than AAMAs.

Furthermore, there are other potential therapeutic approaches targeting the pGlu-Aβ pathway. As seen in Fig. 1, there are multiple steps involved in pGlu-Aβ formation that may be targeted pharmacologically to reduce its formation. Dipeptidyl peptidase-4 (DPP-4) inhibitors including the DPP-4 inhibitor class of glyptins (e.g., sitagliptin, vildagliptin, linagliptin), are treatments approved for the treatment of type 2 diabetes. DPP-4 inhibitors are advantageous in that they have already been shown to be generally well tolerated and safe for long term use [41]. They have begun to attract some research interest in their effects on attenuating cognitive decline in those with both diabetes and MCI or AD [41–44]. In addition, DPP-4 acts to inhibit glucagon-like peptide-1 (GLP-1) potentially having a negative downstream effect on this target which is currently being investigated with GLP-1 agonists for the treatment of AD in two phase 3 clinical trials: evoke (NCT04777396) and evoke+ (NCT04777409) [45]. A large multicenter, randomized, controlled clinical trial (NCT05313529) is currently underway to compare the effects of liraglutide, empagliflozin, and linagliptin on the cognitive function of diabetic patients with MCI.

There are currently no clinical studies investigating meprin β or aminopeptidase inhibitors in AD. In a transgenic APP/lon mouse model of AD, meprin β knockout has been shown to improve cognitive ability and rescue learning behavior impairments [46]. Similarly, aminopeptidase inhibitors have been shown to reduce learning and memory deficits in 3×Tg-AD mice [47]. While aminopeptidase inhibitors have been investigated clinically as potential treatments for hypertension and heart failure, there are currently no clinical candidates for AD[48, 49].

There are also N-Truncated Amyloid Peptide Antibodies (TAPAS) that selectively target the N-terminus of pGlu-Aβ monomers and bind them. These have been shown to reduce amyloid plaque load and rescue memory deficits [50–53]. The TAP01 humanized monoclonal antibody may have advantageous bioavailability with less potential for dose limiting side effects [53]. No clinical studies have yet been performed with these TAPAS which may add another therapeutic target for monoclonal antibodies directed at pGlu-Aβ.

The VIVA-MIND trial utilizes an efficient phase 2A-2B design to establish optimal dosing, safety, tolerability, and efficacy of varoglutamstat in early AD. Phase 2A includes adaptive determination of the highest safe and well-tolerated dose within 3 sentinel dose cohorts “to play the winner”, allowing phase 2B to test the selected dose for the remainder of the trial. We expect a minimum 12 months of time saved by this seamless design compared to time required for two separate phase 2 trials. Methodologically, use of an exact continuous Pocock futility boundary allows for a minimal phase 2A sample size with maximal statistical efficiency.

There are some potential limitations inherent with the VIVA-MIND study design. The adaptive dose decision is operationally complex, as it requires active statistical oversight of a safety boundary, which if breached requires resupply of investigational product to all participants along a short timeline.

The trial is aiming to recruit a diverse participant sample including persons from historically underrepresented and minoritized groups. It is utilizing CSF Elecsys® assays, including Aβ and p-tau measures, with increasing recognition of some of the unique patterns in underrepresented minority populations. For example, CSF Aβ_1-42_ and p-tau-181 are reported to be lower in Black as compared to White individuals [54–56]; however, per study protocol, thresholds for positive results are unadjusted as there were insufficient data to support an alternativeapproach.

Varoglutamstat can address multiple pathogenic contributors to the disease cascade with a single small molecule treatment and has potential for synergistic or additive treatment effects in early AD. The VIVA-MIND study targets early AD with the goal of improving cognition and everyday function and attenuating longer term disease progression through varoglutamstat’s dual pathway mode of action. As of August 2023, 73 participants have been randomized and the study is ongoing according toprotocol.

## AUTHOR CONTRIBUTIONS

Howard H. Feldman (Conceptualization; Investigation; Methodology; Writing – original draft; Writing – review & editing); Karen Messer (Conceptualization; Formal analysis; Methodology; Writing – review & editing); Yuqi Qiu (Formal analysis; Methodology; Writing – review & editing); Marwan Sabbagh (Methodology; Writing – review & editing); Douglas Galasko (Methodology; Writing – review & editing); R. Scott Turner (Methodology; Writing – review & editing); Oscar Lopez (Methodology; Writing – review & editing); Amanda Smith (Methodology; Writing – review & editing); January Durant (Writing – original draft; Writing – review & editing); Jody-Lynn Lupo (Writing – original draft; Writing – review & editing); Carolyn Revta (Project administration; Writing – review & editing); Archana Balasubramanian (Project administration; Writing – review & editing); Kerstin Kuehn-Wache (Writing – review & editing); Tanja Wassmann (Writing – review & editing); Sylvia Schell-Mader (Methodology; Writing – review & editing); Diane M. Jacobs (Methodology; Writing – review & editing); David P. Salmon (Methodology; Writing – review & editing); Gabriel Léger (Methodology; Writing – review & editing); Mari L. DeMarco (Methodology; Writing – review & editing); Frank Weber (Conceptualization; Investigation; Methodology; Writing – review & editing).

## Supplementary Material

Supplementary Material
