# Supplementary material for: Varoglutamstat: Inhibiting Glutaminyl Cyclase as a Novel Target of Therapy in Early Alzheimer’s Disease
Source: J Alzheimers Dis. 2024 Oct 18;101(Suppl 1):S79–93. doi: 10.3233/JAD-231126 (PMC11494639; doi:10.3233/JAD-231126)
Supplement: Supplementary Material [file jad-101-jad231126-s001.pdf]

Supplementary Material

Varoglutamstat: Inhibiting Glutaminyl Cyclase as a Novel Target of Therapy in Early Alzheimer's Disease

Schedule of Events

Visit Number	1	2	3	4	5	6	7	8	9	10	11 EOT/Early Term	12 Post Tx Safety Follow Up
**Study Visit Time Point**	Screening (-90 d)	Baseline (Week 0)	Wk 4 (±7 d)	Wk 8 (±7 d)	Wk 12 (±7 d)	Wk 16 (±7 d)	Wk 24 (±7 d)	Wk 36 (±7 d)	Wk 48 (±7 d)	Wk 60 (±7 d)	Wk 72 (±7 d)	Wk 76 (±7 d)
Informed Consent	X											
Eligibility Review	X	X										
Randomization		X										
Medical History/Demographics	X											
Modified Hachinski Ischemic Scale	X											
Weight & Height	X	X	X	X	X	X	X	X	X	X	X	X
Physical Examination	X	X	X	X	X	X	X	X	X	X	X	X
Neurological Examination	X											
Vital Signs	X	X	X	X	X	X	X	X	X	X	X	X
Concomitant Medication	X	X	X	X	X	X	X	X	X	X	X	X
Adverse Events	X	X	X	X	X	X	X	X	X	X	X	X
12-lead ECG (resting)	X						X				X	
B12 and folate (blood tests)	X											
Clinical Safety Blood Tests	X	X	X	X	X	X	X	X	X	X	X	X
Hemoglobin A1c (HbA1c)	X					X	X		X		X	X
Testosterone (blood test)	X					X	X		X		X	X
Thyroid Function (TSH, T4, T3)	X					X	X		X		X	X
Urinalysis	X	X		X		X	X	X	X		X	X
Infectious Disease Serology	X											
APOE (blood test)		X										
HLA (blood test)		X										
CYP2C19 (blood test)		X										
PrecivityAD® biomarker assays	X											
Blood Collection for Biobanking (blood)	X	X	X	X		X	X		X		X	
Blood Collection for PQ912 levels and QC activity[8]			X	X		X	X		X		X	
Blood Collection for QC in Serum	X		X	X		X	X		X		X	

**Visit Number**	1	2	3	4	5	6	7	8	9	10	11 EOT/Early Term	12 Post Tx Safety Follow Up
**Study Visit Time Point**	Screening (-90 d)	Baseline (Week 0)	Wk 4 (±7 d)	Wk 8 (±7 d)	Wk 12 (±7 d)	Wk 16 (±7 d)	Wk 24 (±7 d)	Wk 36 (±7 d)	Wk 48 (±7 d)	Wk 60 (±7 d)	Wk 72 (±7 d)	Wk 76 (±7 d)
Cranial MRI	X						X				X	
Lumbar Puncture (LP) for CSF biomarkers	X						X				X	
Post-LP Safety Telephone	X						X				X	
C-SSRS	X	X	X	X	X	X	X	X	X	X	X	
MoCA	X						X		X		X	
MMSE	X						X		X		X	
CDR	X				X		X		X		X	
ADAS-Cog13		X			X		X	X	X	X	X	
FAQ		X					X		X		X	
ABC - Category Fluency		X					X		X		X	
ABC - Trail Making Test A & B		X					X		X		X	
ABC - Digit Symbol Substitution		X					X		X		X	
ABC - Boston Naming Test		X					X		X		X	
ABC – RAVLT		X					X		X		X	
ABC – Number Span		X					X		X		X	
NPI		X					X		X		X	
Quantitative EEG		X					X		X		X	
Research Satisfaction Survey		X					X		X		X	
Dispense Study Drug		X	X	X	X	X	X	X	X	X		
Study Drug Instruction Phone Call			X									
Study Drug Accountability			X	X	X	X	X	X	X	X	X	
Treatment Blinding Questionnaire											X	

ECG, electrocardiogram; QC, glutaminyl cyclase; MRI, Magnetic Resonance Imaging; CSF, cerebrospinal fluid; LP, lumbar puncture; MoCA, Montreal Cognitive Assessment; MMSE, Mini-Mental State Examination; CDR, Clinical Dementia Rating; ADAS-Cog13, Alzheimer's Disease Assessment Scale-Cognitive Subscale – 13 Item; FAQ, Functional Activities Questionnaire; ABC, ADNI Battery Composite; RAVLT, Rey Auditory Verbal Learning Test; NPI, Neuropsychiatric Inventory; EEG, electroencephalogram; C-SSRS, Columbia-Suicide Severity Rating Scale
